# Prognostic value of tumor mutation burden in patients with advanced gastric cancer receiving first-line chemotherapy

**DOI:** 10.3389/fonc.2022.1007146

**Published:** 2023-01-04

**Authors:** Xiao-Peng Duan, Ke Liu, Xiao-Dong Jiao, Bao-Dong Qin, Bing Li, Xi He, Yan Ling, Ying Wu, Shi-Qi Chen, Yuan-Sheng Zang

**Affiliations:** ^1^ Department of Medical Oncology, Changzheng Hospital, Naval Medical University, Shanghai, China; ^2^ Burning Rock Biotech, Shanghai, China

**Keywords:** gastric cancer, chemotherapy, tumor mutation burden, biomarker, prognostic value, mechanism

## Abstract

**Background:**

Tumor mutation burden (TMB) is a promising biomarker positively associated with the benefit of immunotherapy and that might predict the outcome of chemotherapy. We described the prognostic value of TMB in advanced gastric cancer and explored the underlying mechanism.

**Methods:**

We enrolled 155 TMB-evaluated advanced gastric cancer patients and analyzed the relationship between clinicopathological characteristics and both overall survival (OS) and progression-free survival (PFS) among 40 patients treated with first-line chemotherapy. We further verified the distribution of TMB and analyzed the potential mechanism underlying the prognosis based on The Cancer Genome Atlas (TCGA) database.

**Results:**

Among the 155 patients, 29 (18.7%) were TMB-high (TMB ≥ 10), roughly the same as the proportion in the TCGA data. Of the 40 patients receiving first-line chemotherapy, the median OS (7.9 *vs*. 12.1 months; HR 3.18; *p* = 0.0056) and PFS (4.4 *vs*. 6.2 months; HR 2.94; *p* = 0.0099) of the tissue-tested TMB (tTMB)-high patients were inferior to those of the tTMB-low patients. Similarly, unfavorable median OS (9.9 *vs*. 12.1 months; HR 2.11; *p* = 0.028) and PFS (5.3 *vs*. 6.5 months; HR 2.49; *p* = 0.0054) were shown in the blood-tested TMB (bTMB)-high than in the bTMB-low patients. The Cox analysis demonstrated that both tTMB-high and bTMB-high were significant independent predictors of dreadful OS and PFS. The differentially expressed genes (DEGs) according to TMB status were most significantly enriched in the downregulated metabolic pathway among the TMB-high patients.

**Conclusions:**

TMB-high advanced gastric cancer patients accounted for around one-sixth and had a poorer prognosis than TMB-low patients when treated with first-line chemotherapy. The potential mechanism might be the downregulated metabolic activity in TMB-high patients.

## Introduction

According to the latest global cancer data in 2020, gastric cancer is the fifth most common malignant tumor (1,089,103 cases, 5.6% of total cases) and the fourth leading cause of deaths (768,793 deaths, 7.7% of the total) around the world (1). Although curative surgical resection is the most effective treatment for improved prognosis, approximately 50% of patients have distant metastasis, with this rising to more than 80% in China, meaning patients lose the opportunity at the time of diagnosis (2–4). Therefore, systemic therapy, especially chemotherapy, is routinely performed for advanced cases (4). However, due to the high degree of heterogeneity in advanced gastric cancer (4–6), there have been few optimal assessable biomarkers to generally characterize the patients and guide the formulation of the treatment plans.

With the rapid development of precision medicine, massive gene expression and mutation information ascertained *via* next-generation sequencing (NGS) are reforming the treatment strategies of malignancy (7). Excavating the meaning of these abundant data is of critical importance in decomposing the heterogeneity and in optimizing the therapeutic regimen (4, 8). Tumor mutation burden (TMB), representing the ability of tumors to produce new antigens, is one of the screening methods derived from NGS (9, 10). TMB has been recognized as a promising biomarker positively associated with immunotherapy with improved benefits for a variety of tumors (11–15), including gastric cancer (16–18). However, the indications for immunotherapy only cover a narrow range, and chemotherapy is still the cornerstone in the first-line treatment of advanced gastric cancer. Whether TMB could predict clinical outcomes in advanced gastric cancer patients treated with chemotherapy is still unclear.

The main purpose of this study was to evaluate the prognostic value of TMB on overall survival (OS) and progression-free survival (PFS) in advanced gastric cancer patients treated with first-line chemotherapy. The data from The Cancer Genome Atlas (TCGA) were further analyzed to verify the distribution of TMB and explore the potential biologic mechanism underlying the prognosis.

## Methods

### The Chinese cohort

We enrolled 155 TMB-evaluated advanced gastric cancer patients in our department from December 2017 to June 2020. Among these patients, 40 accepted first-line chemotherapy with both tissue and blood specimens tested by NGS. All patients were histologically confirmed with recurrent or metastatic malignancy of stomach adenocarcinoma and microsatellite stability (MSS) status sequenced by NGS. We use the eighth edition of the TNM staging system of gastric cancer established by the American Joint Committee on Cancer to define the pathological stage. The response assessment was evaluated by the associate chief physician on the basis of the Response Evaluation Criteria in Solid Tumors (RECIST) version 1.1 after treatment. The human epidermal growth factor receptor-2 (HER2) status was detected by immunohistochemistry (IHC) and fluorescence in-situ hybridization (FISH). Cases with IHC 3+ were directly judged as HER2 positive, and cases with IHC 1+ and IHC 0 were HER2 negative. The cases of IHC 2+ are uncertain cases, which need the FISH test to finally determine the HER2 status. The IHC 2+ cases with and without HER2 amplification were HER2 positive and negative, respectively. All HER2-positive patients received anti-HER2 treatment in the first line. Survival and disease status were affirmed by reviewing the patients’ medical records and through telephone follow-up visits. Both tumor biopsy and blood sample were tested by NGS. The quality and size of the fragments were assessed by high sensitivity DNA kit using Bioanalyzer 2100 (Agilent Technologies, CA, USA). Indexed samples were sequenced on NextSeq 500 (Illumina, Inc., CA, USA) with paired-end reads and average sequencing depth of 1,000× for tissue samples and 10,000× for liquid biopsy samples. The detailed procedure of NGS library building, sequencing, and data analysis was conducted based on a previous study (19). TMB was defined as the density of non-synonymous mutations in the protein-coding region. The non-synonymous mutations that lead to variations of amino acids represented the ability of new antigen generation. The counted mutations included missense, nonsense, indels, and frameshift mutations. The TMB value equals the amount of non-synonymous mutation sites divided by the base pairs of the sequencing coding region (a panel of 295 genes spanning 0.98 Mb, a panel of 520 genes spanning 1.26 Mb), taking the mutation amount per megabase pairs as a unit. This study was approved by the Institutional Ethics Committee of The Second Affiliated Hospital of Naval Medical University.

### The TCGA cohort

Information regarding stage III–IV stomach adenocarcinoma was downloaded from the TCGA database. The gene mutation data were downloaded in mutation annotation format (MAF) file with VerScan2 Variant Aggregation and Masking workflow type. We visualized the somatic gene mutation characteristics by using the “maftools” package in R software. TMB was equal to the number of somatic mutations presented in MAF file divided by the total length of the exons (around 38 Mb). The transcriptome profiling was downloaded in the HTSeq-FPKM workflow type. We identified the differentially expressed genes (DEGs) between the TMB-high and the TMB-low groups by using the “limma” package in R software. The DEGs with |log(fold change)| >1 and p-value <0.05 were selected for subsequent analysis. We visualized the gene differential expression related to TMB status by using the “pheatmap” R package. Furthermore, we conducted Gene Ontology (GO), Kyoto Encyclopedia of Genes and Genomes (KEGG), and gene set enrichment analysis (GSEA) functional pathway enrichment analyses to explore the biological mechanism underlying the prognosis and to visualize the results. We also analyzed the relationship between TMB status and immune cell infiltration according to the “CIBERSORT” R package.

### Statistical analysis

We described the distribution of TMB and gene mutation characteristics in advanced gastric cancer of the above two cohorts by dividing the patients into two groups according to TMB status: TMB-high (TMB ≥ 10) and TMB-low (TMB < 10). The cutoff value of TMB was determined on the basis of the KEYNOTE-158 clinical trial results approved by The Food and Drug Administration (20, 21), which was the same as the trial KEYNOTE-062 for gastric cancer with pembrolizumab ± chemotherapy as first-line treatment (22). We compared the OS and PFS in patients treated with first-line chemotherapy according to TMB status. The OS was defined as the duration from systemic therapy initiation to the last follow-up visit or death. The PFS was estimated from the beginning of treatment to the date of disease progression. The OS and PFS were analyzed by the Kaplan–Meier method, in which the *p*-value was evaluated by the log-rank test. The prognostic factors of OS and PFS were evaluated by the Cox proportional hazards model. The baseline characteristics included in the analyses were tTMB, bTMB, age, gender, BMI (<18.5 or ≥24 vs. 18.5-23.9), histological type (undifferentiated vs. differentiated), primary site (gastric fundus and body vs. gastric horn and antrum), number of metastatic organs (<3 vs. ≥3), presence of peritoneal metastasis (yes vs. no), previous surgery (yes vs. no), local therapy (yes vs. no), tumor marker level (elevated vs. normal), HER2 immunohistochemistry status (positive vs. negative), and erythroblastic leukemia viral oncogene homolog 2 (ERBB2) genotype (mutant vs. wild).

The difference in genetic and clinical characteristics between the two groups was analyzed by the chi-square test for categorical variables and by the Mann–Whitney *U* test for continuous variables. All the data analyses and visualization were conducted using SPSS Statistics 25, R 4.1.1, and GraphPad Prism 9 software. All recorded tests were two-tailed and a *p*-value <0.05 was considered statistically significant.

## Results

### Patients’ characteristics

**Figure 1:**
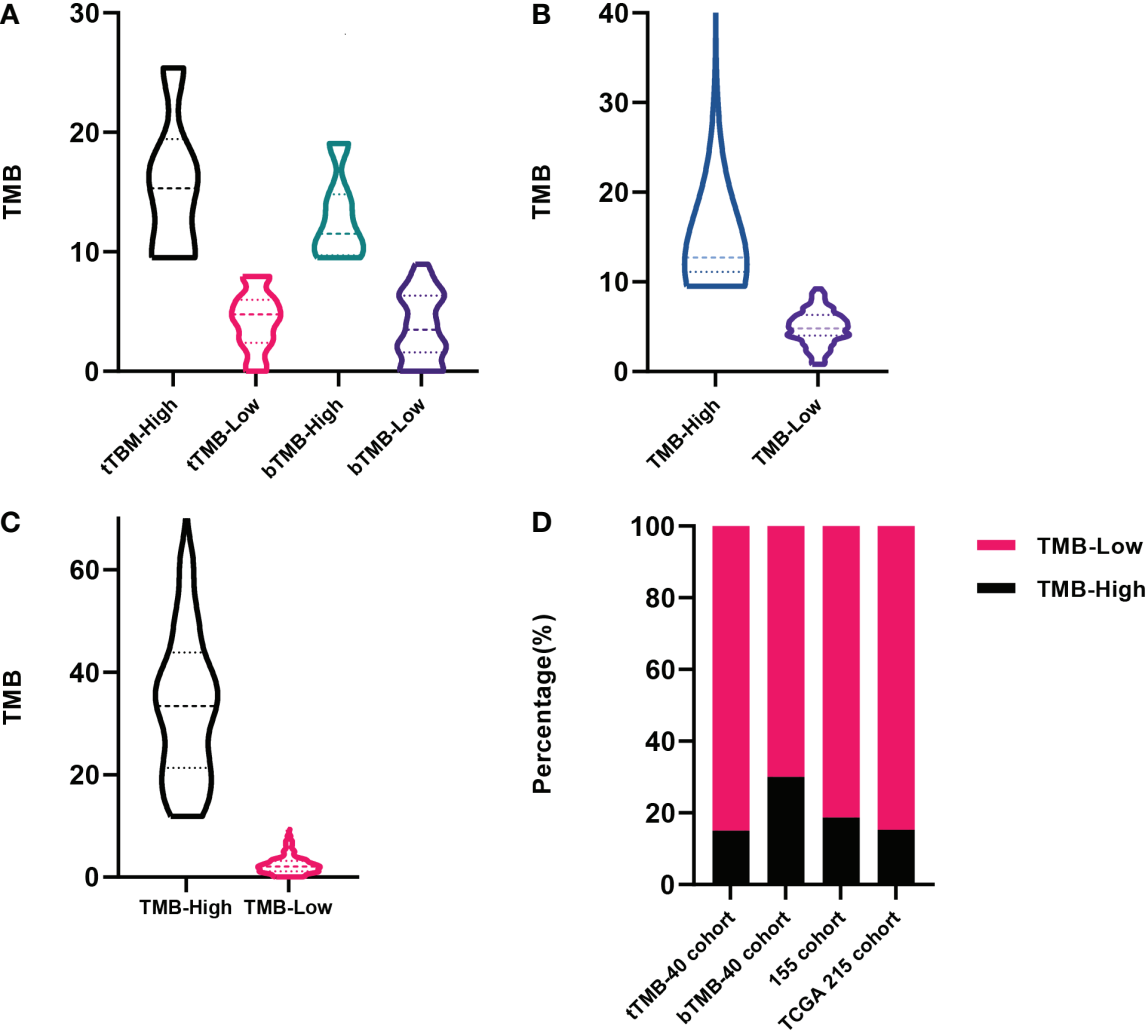
The distribution of tumor mutation burden (TMB) among patients. **(A)** Forty patients receiving first-line chemotherapy in our cohort tested by tissue samples and blood samples. **(B)** Patients included in our 155 cohort. **(C)** Patients recorded in The Cancer Genome Atlas (TCGA) database. **(D)** The proportion of patients among the above cohorts according to TMB status.

**Figure 2:**
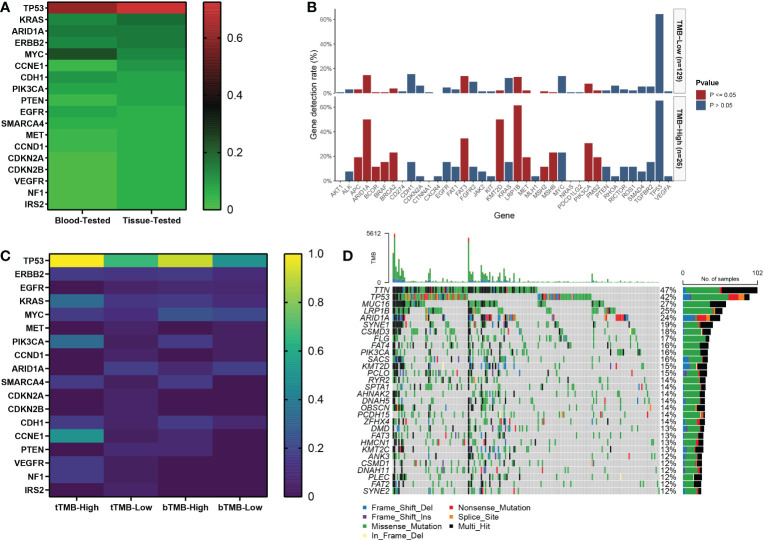
The gene mutation spectrum of patients with advanced gastric cancer in our cohort. **(A)** The mutation frequency of tissue-tested and blood-tested samples of 18 genes with mutation frequency equal to or higher than 5% in 40 patients receiving first-line chemotherapy. **(B)** The association between TMB status and gene mutation in 155 patients. **(C)** The gene mutation frequency heatmap according to TMB status of tissue and blood samples in 40 patients. **(D)** Waterfall of gene mutations in the TCGA cohort.

Of the 40 patients receiving first-line chemotherapy, 16 (15.0%) were tTMB-high and 24 (75.0%) were tTMB-low. The median TMB values of tTMB-high and tTMB-low patients were 15.3 and 4.8, respectively. Twelve (30.0%) patients were bTMB-high and 28 (70.0%) were bTMB-low. The median TMB values of the bTMB-high and bTMB-low patients were 11.5 and 3.5 (**Figure 1A**). The clinicopathological and genetic characteristics of the patients receiving chemotherapy according to TMB status are listed in **Table 1**. The difference between the other clinicopathological factors was not statistically significant. To demonstrate the effect of the tested sample type, we selected 18 genes with a mutation frequency ≥5% to state the gene mutation spectrum. There was no significant difference in gene mutation frequency between the blood-tested and the tissue-tested samples (**Figures 2A, C**).

**Table 1:** Clinicopathological characteristics of advanced gastric cancer patients receiving first-line chemotherapy.

Characteristics	Tissue-tested			Blood-tested	
	TMB-high (*n* = 6)	TMB-low (*n* = 34)		TMB-high (*n* = 12)	TMB-low (*n* = 28)
Age (years)					
Median (range)	57 (34-82)	56 (33-77)		59 (34-82)	55.5 (33-77)
Gender					
Male	5 (83.3)	18 (52.9)		7 (58.3)	16 (57.1)
Female	1 (16.7)	17 (47.1)		5 (41.7)	12 (42.9)
BMI					
<18.5 or >=24	2 (33.3)	16 (47.1)		4 (33.3)	14 (50.0)
18.5-23.9	4 (66.7)	18 (52.9)		8 (66.7)	14 (50.0)
Primary site					
Gastric fundus and body	4 (66.7)	16 (47.1)		7 (58.3)	16 (57.1)
Gastric horn and antrum	2 (33.3)	18 (52.9)		5 (41.7)	12 (42.9)
Histological					
Undifferentiated	2 (33.3)	6 (17.6)		9 (75.0)	23 (82.1)
Differentiated	4 (66.7)	28 (82.4)		3 (25.0)	5 (17.9)
Number of metastatic organs					
>=3	3 (50.0)	16 (47.1)		7 (58.3)	13 (46.4)
<3	3 (50.0)	18 (52.9)		5 (41.7)	15 (53.6)
Metastasis of peritoneum					
Yes	1 (16.7)	16 (47.1)		3 (25.0)	14 (50.0)
No	5 (83.3)	18 (52.9)		9 (75.0)	14 (50.0)
Previous surgery					
Yes	2 (33.3)	14 (41.2)		5 (41.7)	11 (39.3)
No	4 (66.7)	20 (58.8)		7 (58.3)	17 (60.7)
Local therapy					
Yes	2 (33.3)	10 (29.4)		4 (33.3)	8 (28.6)
No	4 (66.7)	24 (70.6)		8 (66.7)	21 (71.4)
NSE					
Elevated	2 (33.3)	12 (35.3)		3 (25.0)	11 (39.3)
Normal	4 (66.7)	22 (64.7)		9 (75.0)	17 (60.7)
CEA					
Elevated	3 (50.0)	19 (55.9)		7 (58.3)	15 (53.6)
Normal	3 (50.0)	15 (44.1)		5 (41.7)	13 (46.4)
CA199					
Elevated	2 (33.3)	13 (38.2)		4 (33.3)	11 (39.3)
Normal	4 (66.7)	21 (61.8)		8 (66.7)	17 (60.7)
CA125					
Elevated	4 (66.7)	18 (52.9)		8 (66.7)	14 (50.0)
Normal	2 (33.3)	16 (47.1)		4 (33.3)	14 (50.0)
CA724					
Elevated	2 (33.3)	21 (61.8)		7 (58.3)	16 (57.1)
Normal	4 (66.7)	13 (38.2)		5 (41.7)	12 (42.9)
HER2 immunohistochemistry					
Positive	1 (16.7)	5 (14.7)		3 (25.0)	3 (10.7)
Negative	5 (83.3)	29 (85.3)		9 (75.0)	25 (89.3)
Blood-tested ERBB2					
Mutant	1 (16.7)	4 (11.8)		2 (16.7)	3 (10.7)
Wild	5 (83.3)	30 (88.2)		10 (83.3)	25 (89.3)
Tissue-tested ERBB2					
Mutant	1 (16.7)	5 (14.7)		2 (16.7)	4 (14.3)
Wild	5 (83.3)	29 (85.3)		10 (83.3)	24 (85.7)
bTMB	13.57 ± 3.22	5.14 ± 4.39		12.80 ± 3.44	3.67 ± 2.84
tTMB	15.42 ± 5.95	4.30 ± 2.389		10.62 ± 6.58	3.97 ± 2.35

BMI, body mass index; GE, gastroesophageal; NSE, neuron-specific enolase; CEA, carcinoembryonic antigen; CA125, carbohydrate antigen-125; CA724, carbohydrate antigen-724; HER2, human epidermal growth factor receptor 2; ERBB2, erythroblastic leukemia viral oncogene homolog 2; bTMB, blood-tested tumor mutation burden; tTMB, tissue-tested tumor mutation burden.

Among the 155 patients, 29 (18.7%) were TMB-high and 126 (81.3%) were TMB-low. The median TMB values of the TMB-high and the TMB-low patients were 12.7 and 4.8, respectively (**Figure 1B**). There were 13 gene mutations tested by NGS panels with a higher frequency in TMB-high than in TMB-low tumors. The top 3 genes of mutation frequency were *LRP1B*, *ARID1A*, and *KMT2D* (61.5% *vs*. 13.2%, 50.0% *vs*. 14.7%, 50.0% *vs*. 2.3%, respectively) (**Figure 2B**).

Among the 215 patients in the TCGA cohort, 33 (15.3%) were TMB-high and 182 (84.7%) were TMB-low. The median TMB values of the TMB-high and the TMB-low patients were 35.0 and 2.1, respectively (**Figure 1C**). The gene mutation spectrum was visualized in a waterfall map (**Figure 2D**). Three of the top 5 frequency mutated genes (*TP53*, *LRP1B*, *ARID1A*) were the same as those in our cohort of 155 patients.

The three cohorts stated above had a similar proportion of TMB-high patients accounting for around one-sixth (**Figure 1D**).

### Survival analysis

**Figure 3:**
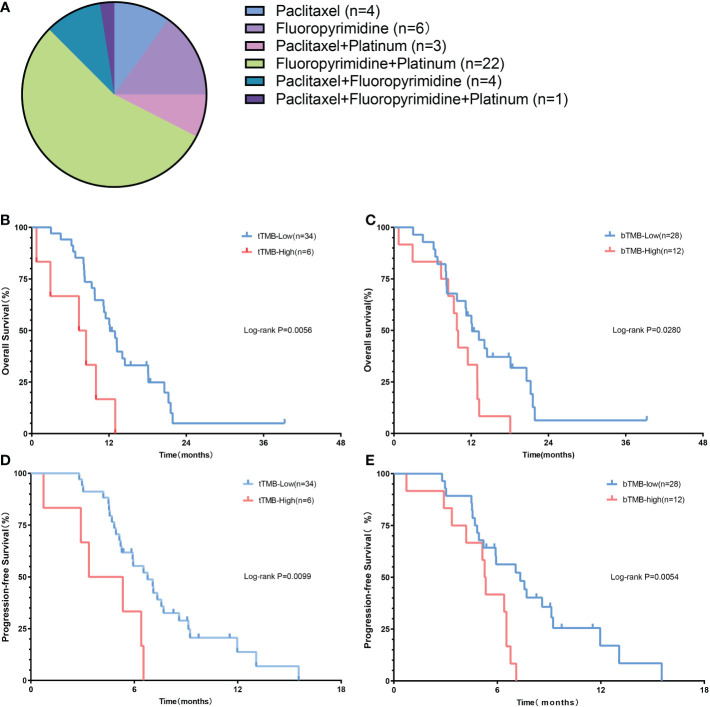
The prognostic value of TMB in advanced gastric cancer patients. **(A)** The regimen of chemotherapy. **(B)** The OS of patients according to tTMB. **(C)** The PFS of patients according to tTMB. **(D)** The OS of patients according to bTMB. **(E)** The PFS of patients according to bTM.

To demonstrate the prognostic value of TMB, we conducted the K-M survival analysis of patients receiving first-line chemotherapy. The detailed regimen of chemotherapy is listed in **Figure 3A**. The median OS of the tTMB-high patients (7.9 months; 95% CI, 2.3-11.8 months) was significantly shorter than that of the tTMB-low (12.1 months; 95% CI, 10.9-15.6 months) according to survival curve comparison (HR 3.18; 95% CI, 0.80-12.7; log-rank *p* = 0.0056) (**Figure 3B**). The median PFS of the tTMB-high patients (4.4 months; 95% CI, 1.8-6.6 months) was also inferior to that of the tTMB-low patients (6.2 months; 95% CI, 5.9-8.0 months) as verified by the survival curve analysis (HR 2.94; 95% CI, 0.77-11.21; log-rank *p* = 0.0099) (**Figure 3D**). Similarly, the bTMB-high patients were associated with poor median OS (HR 2.11; 95% CI, 0.92-4.84; log-rank *p* = 0.028). An unfavorable OS was shown in the bTMB-high patients (9.9 months; 95% CI, 6.8-12.7 months) compared with the bTMB-low patients (12.1 months; 95% CI, 10.6-16.3 months) (**Figure 3C**). bTMB-high was associated with dreadful median PFS as well (HR 2.49; 95% CI, 1.04-5.99; log-rank *p* = 0.0054). A shorter PFS also was confirmed in the bTMB-high patients (5.3 months; 95% CI, 3.8-6.2 months) than in the bTMB-low patients (6.5 months; 95% CI, 5.9-8.4 months) (**Figure 3E**).

### Univariate and multivariate analyses

**Figure 4:**
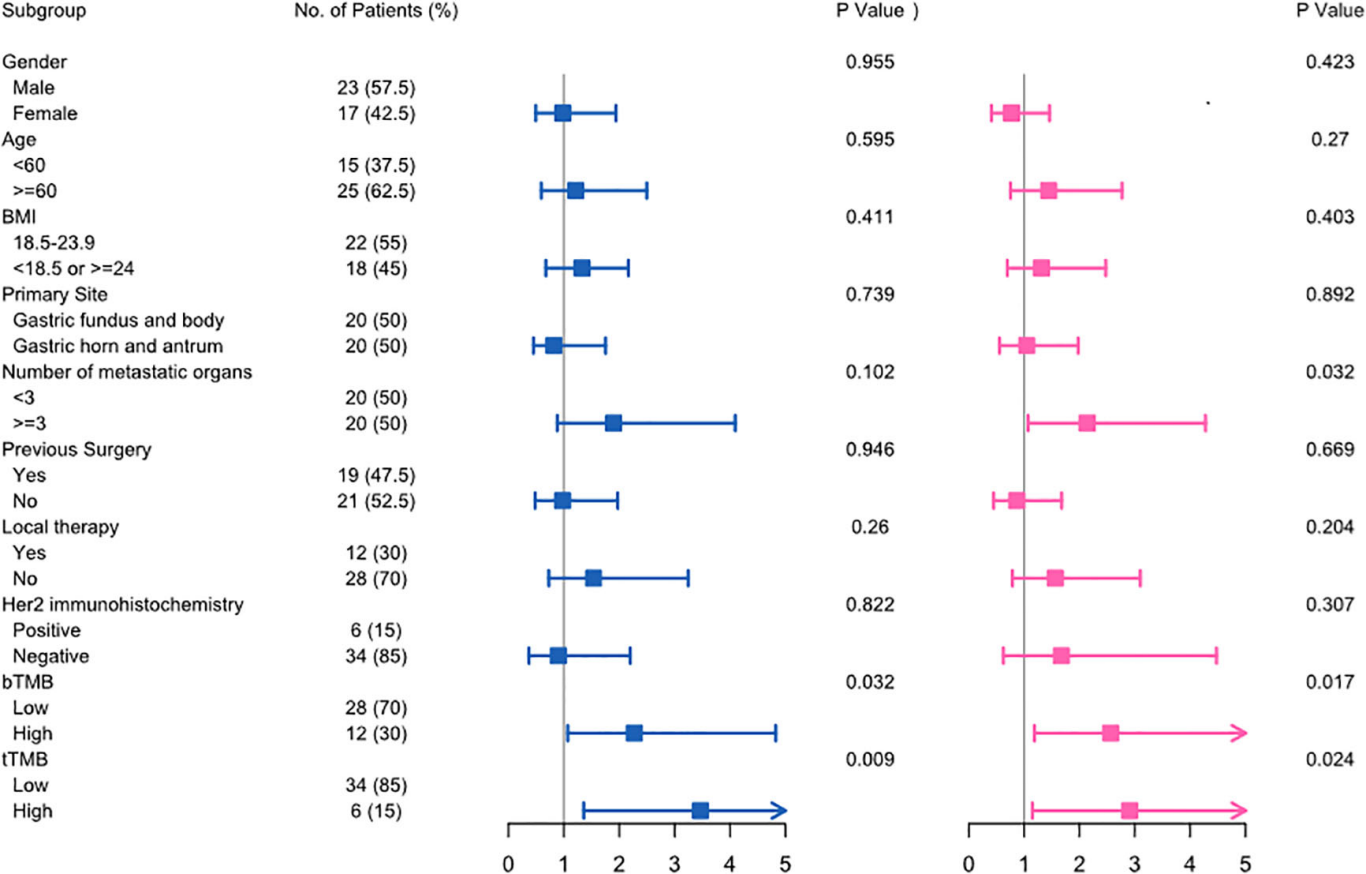
The univariate Cox analysis of clinicopathological factors for overall survival (left) and progression-free survival (right) in advanced gastric cancer.

In the univariate analysis, both tTMB-high (HR 3.47; 95% CI, 1.36-8.83; *p* = 0.009) and bTMB-high (HR 2.27; 95% CI, 1.07-4.83; *p* = 0.032) were associated with unfavorable OS. Similarly, both tTMB-high (HR 2.91; 95% CI, 1.15-7.36; *p* = 0.024) and bTMB-high (HR 2.56; 95% CI, 1.18-5.56; *p* = 0.017) were associated with poor PFS (**Figure 4**). Additionally, the number of metastatic organs (≥3) was also associated with worse PFS. The detailed hazard ratio of each characteristic is provided in **Supplementary Table 1**.

The multivariate analysis further demonstrated that bTMB-high was the prognostic factor of worse OS (*p* = 0.006) and PFS (*p* = 0.013), respectively.

According to the univariate and multivariate analyses, we identified that TMB was an independent predictor of outcome in advanced gastric cancer patients receiving first-line systemic chemotherapy.

### Functional analysis

**Figure 5:**
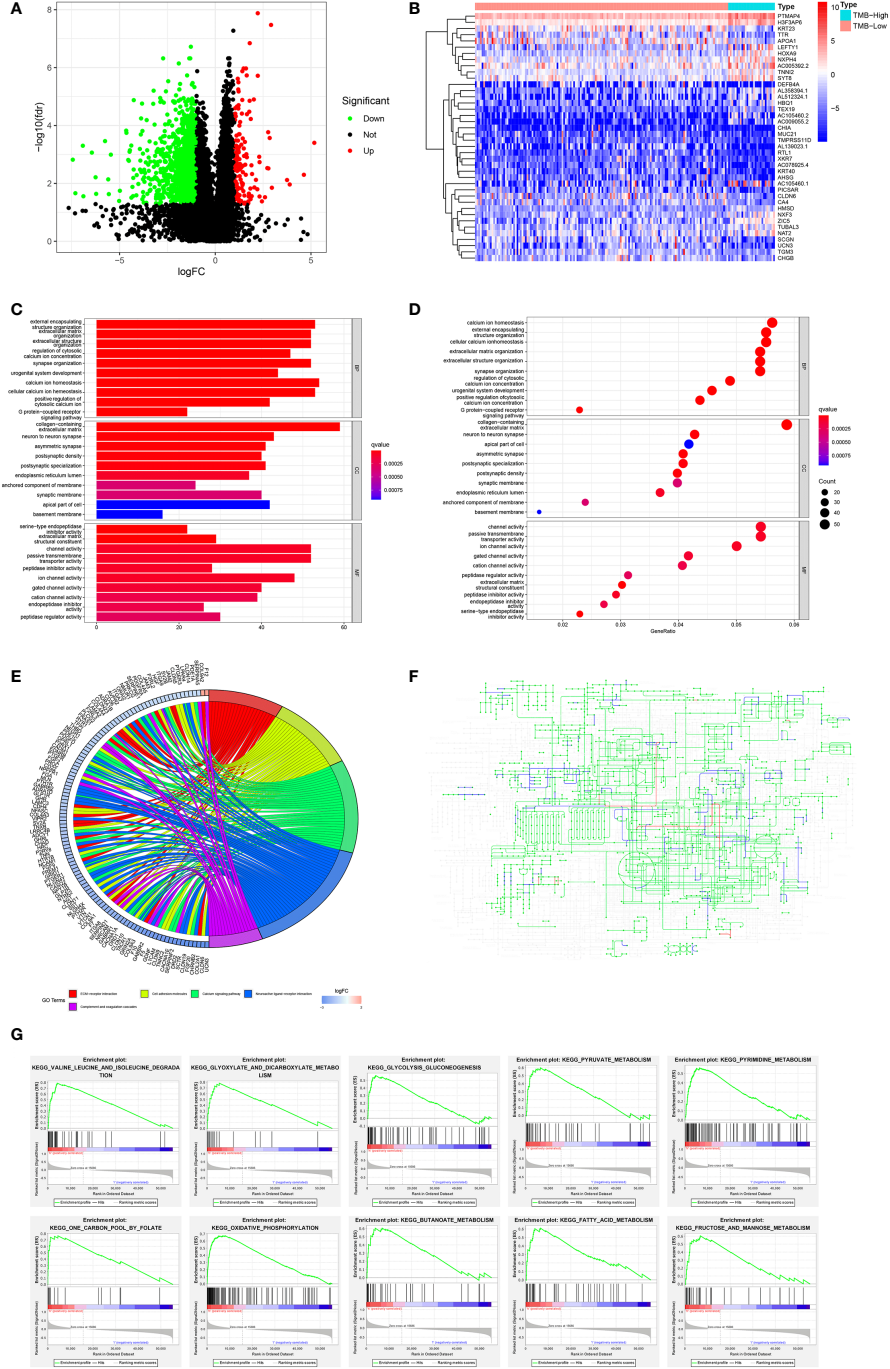
Variation of genes according to TMB status and function analysis in the TCGA cohort. **(A)** Volcano map of DEGs related to TMB status. **(B)** Heatmap of the top 40 DEGs. **(C, D)** GO and **(E)** KEGG analyses of DEGs. **(F)** The upregulated (red) and downregulated (blue) pathways in the KEGG metabolic pathway map of DEGs. **(G)** GESA analysis regarding metabolic pathway.

We divided the TCGA cohort patients into TMB-high and TMB-low groups according to the cutoff value of 10. Based on the transcriptome data of the TCGA, we identified 1,396 DEGs consisting of 1,296 downregulated genes and 100 upregulated genes in the TMB-high group compared with the TMB-low group (**Figure 5A**). The top 40 DEGs were presented in a heatmap (**Figure 5B**). The GO analysis revealed that DEGs were chiefly enriched in ion channel activity and communications with the extracellular environment (**Figures 5C, D**). Similarly, the KEGG analysis indicated that DEGs mainly took part in the calcium signaling pathway, cascade reaction, ECM–receptor interaction, and cell adhesion (**Figure 5E**). In the pathway analysis, the DEGs enriched in the metabolic pathway consisting of 86 mutated genes were mostly downregulated (**Figure 5F**). In addition, we conducted GSEA functional analysis and found that 10 of the top 20 pathways were involved in the metabolic pathway (**Figure 5G**).

**Figure 6:**
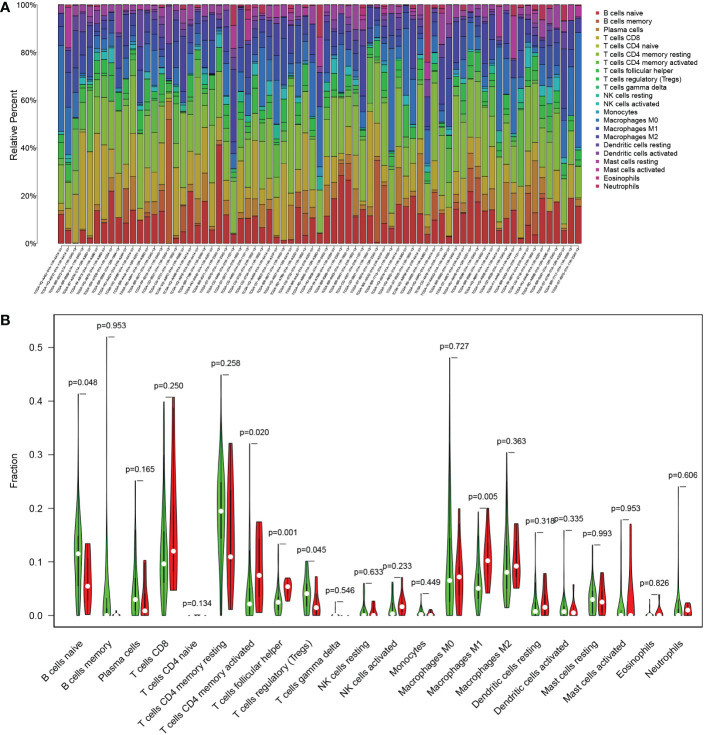
The immune cell infiltration related to TMB status in the TCGA cohort. **(A)** Proportion of 22 infiltrating immune cells in each sample. **(B)** Difference of infiltrating immune cells between the TMB-high (red) and the TMB-low (green) groups.

Furthermore, we analyzed the immune cell infiltration among the TCGA patients since TMB was reported as an immunotherapy biomarker. Except for T-cell follicular helper and macrophage M1, there was no significantly different infiltration of immune cells between the TMB-high and the TMB-low groups (**Figures 6A, B**).

In summary, the downregulation of metabolic activity might be the mechanism underlying the resistance to chemotherapy in TMB-high patients.

## Discussion

We have confirmed that TMB status could characterize advanced gastric cancer, and around one-sixth of the patients were TMB-high in our study. The OS and PFS of the TMB-high patients receiving first-line chemotherapy were evidently shorter than those of the TMB-low patients. Furthermore, the univariate and multivariate analyses verified that bTMB-high was an independent poor prognostic factor. Different treatments would affect the efficacy and, thus, blur the predictive value of TMB. The baseline proportion of patients receiving previous surgery, anti-HER2 therapy, and local therapy in the TMB-high and the TMB-low groups had no statistical difference in our study. Moreover, previous studies had shown that mismatch repair (MMR) status has a certain correlation with TMB and immunotherapy efficacy (23). The patients enrolled in our study all had MSS status. Furthermore, EBV(+) is one of the molecular subtypes of TCGA for gastric cancer, but the positivity rate of EBV status for advanced gastric cancer is low. The predictive value of EBV status for the immunotherapy efficacy of gastric cancer is significantly correlated with the expression of PD-L1 (24). The value of EBV status is unknown, lacking prospective large sample clinical study validation. Therefore, the EBV test is not routinely recommended in the guidelines, and we did not include EBV status in our analysis. Above all, the setting of inclusion criteria and the control of baseline variables were adopted to avoid the effect of confounding factors on the prognosis prediction of TMB.

TMB first came into being as a representative immunotherapy biomarker. It was defined as the sum of non-synonymous mutations that led to new antigen production. The tumors presenting with more new antigens were more likely to be recognized by the immune cells. According to the analysis of whole exon sequencing (WES) data of patients treated with CTLA-4 antibody, the researchers first found out the relationship between TMB and immunotherapy efficacy (25), and then a series of CheckMate clinical trials demonstrated that patients with TMB-high had a better first-line immunotherapeutic response and progression-free survival (26–28). In gastric cancer, immune monotherapy was permitted to be used for the second-line treatment of TMB-high patients in the 2020.1 version gastric cancer clinical guidelines of the National Comprehensive Cancer Network (NCCN) based on the remarkable therapeutic effect of the KEYNOTE-158 clinical trial (20). Recently, the FDA has officially approved PD-1 inhibitors (Opdivo, nivolumab) combined with chemotherapy as the first-line therapy for advanced gastric cancer patients according to the results of the CheckMate-649 trial (29). Meanwhile, the FDA granted accelerated approval to PD-1 inhibitors (Keytruda, pembrolizumab) combined with trastuzumab and chemotherapy for the first-line therapy of HER2-positive advanced gastric carcinoma in accordance with the mid-term data of the KEYNOTE-811 trial (30). Although immunotherapy is in full swing now, chemotherapy is still the cornerstone in the first-line therapy of advanced gastric carcinoma. Except for several single proteins (31, 32), there has been no assessable clinical biomarker with prognostic value on chemotherapy in advanced gastric carcinoma. In our study, using TMB as a novel biomarker we divided patients into two groups and presented different clinical outcomes when treated with first-line chemotherapy. As the predictive function of TMB in immunotherapy has been confirmed, clarifying the prognostic value of TMB on chemotherapy is of great importance in optimizing the choice of first-line treatment in advanced gastric cancer.

Aside from advanced cases, TMB was also related to the prognosis of resectable gastric carcinoma. Partially different from our results, the OS of TMB-high patients was superior to that of TMB-low patients in resectable cases (33, 34). However, TMB-low patients receiving postoperative chemotherapy or chemoradiotherapy had a better clinical benefit of OS and disease-free survival (DFS) in resectable gastric carcinoma (34), which was similar to advanced patients treated with first-line chemotherapy in our study. These results further strengthened the evidence linking TMB with chemotherapy. Wang et al. reckoned that the poor response to postoperative chemotherapy or chemoradiotherapy in TMB-high patients might be related to TMB-associated NK cell infiltration and hypoxia microenvironment (34). Due to limited research on the relevance between TMB and chemotherapy in gastric cancer, the definite mechanism remains incompletely illuminated. Based on our experience with lung cancer, DNA damage response and repair system might be the key point of this effect (35, 36). In our study, there were 11 gene mutations with different frequencies according to TMB status, mainly distributed in pathways related to DNA repair system and tumor proliferation including proto-oncogenes and tumor suppressor genes. *LRP1B* and *ARID1A* both were tumor suppressor genes with a higher mutation frequency in more than 50% of TMB-high patients. *LRP1B* was the highest frequency mutated gene of TMB-high patients in our study and also had been validated to be associated with a high level of TMB in various types of cancer in previous studies (37–39). Interestingly, a multicenter pan-cancer research showed that patients with *LRP1B* mutation had a better outcome with immunotherapy, independent of TMB status (40). A previous study also demonstrated that *ARID1A* as a subunit of the chromatin remodeling complex was the second most frequently mutated gene following *TP53* mutation in gastric cancer (41). Similarly, *ARID1A* mutation ranked third in our results. Although without statistically significant difference according to TMB status, *TP53* was still the most common mutation gene in advanced gastric cancer in our study, which was in accordance with previous studies (41, 42). Because there are still some contradictions between studies, further fundamental and clinical research is required to clarify the biological pathways of TMB in advanced gastric cancer.

Unsatisfactorily, except for the unclear biological mechanism, the representative detection methods of TMB also remain controversial (43). In order to enhance the credibility of the results, we evaluated TMB based on the sequencing results of both blood and tissue samples. Both tTMB-high and bTMB-high were significant independent predictors of poor OS and PFS in our study. Moreover, tTMB had a higher predictive value for OS than bTMB. At present, tTMB detection is mainly based on tumor tissue biopsy. Tissue biopsy is still the gold standard for tumor diagnosis and prognosis (44), which can be confirmed by our research. However, the practicability of tTMB usually depends on the location of the tumor and the performance status of the patients, especially in advanced tumor. Even if tissue biopsy can be performed, about 30% of patients cannot provide enough tissue for NGS detection due to the many clinical tests (43). Compared with blood samples, tumor biopsy limited to a specific area could not accurately reflect the whole tumor mutation panorama over a period of time, especially in advanced gastric cancer with a high degree of spatial and temporal heterogeneity (45). Researchers have corroborated that bTMB tested by circulating tumor DNA (ctDNA) of blood samples could be accurately and repeatedly measured and effectively predict the immunotherapeutic effect (46, 47). Similar to our results, the prediction of bTMB on PFS was more accurate than tTMB in patients treated with first-line chemotherapy. bTMB might reflect the immediate situation of the whole body tumor burden and have better predictive values in the short term. To date, 35 studies focusing on ctDNA as a diagnostic tool, prognostic marker, or standard measure of tumor heterogeneity in gastrointestinal cancer are widely developed (48, 49). Plasma ctDNA has been validated to accurately monitor the efficacy of chemotherapy in advanced gastric cancer. With the maturity of detection technology and evaluation criterion, more meaningful findings related to bTMB are expected in the future.

As for the biological mechanism of poor prognosis, the KEGG and GSEA analyses both indicated that the downregulation of metabolic activity might be the reason. As we know, the efficacy of chemotherapy is mainly based on the rapid proliferation and the high metabolism of tumor cells. Researchers had demonstrated that low metabolic activity such as quiescence or stationarity could help tumors escape from chemotherapeutic drugs that usually target cells with rapid proliferation, which was consistent with our results. The hypometabolic status of cancer cells is considered a therapeutic challenge because it induces tumors into a dormant state, thus avoiding the inherent antitumor monitoring system and being resistant to cytotoxic therapy including chemotherapy and radiotherapy (50). Furthermore, cancer stem cells (CSCs) could possibly be the key components in this hypometabolic status maintenance (50–53). The induction of cell cycle arrest at the G2/M phase or G0–G1 transition might be an important process for CSCs to enter into a hypometabolic status (54, 55). Meanwhile, we did not observe a difference in immune killer cell infiltration such as effective T cells according to TMB status.

However, the biggest limitation of our study is that the sample capacity is relatively small. Although we enrolled 155 patients, only 40 of them presented with intact survival and genetic data that met the criterion to analyze the prognosis of TMB on chemotherapy. Therefore, further large-scale studies need to be conducted to validate our results. Another limitation is that we explored the mechanism by using the transcriptome information from the TCGA database since we lacked RNA-sequencing data. We need to establish our own gene expression database to excavate the mechanism of different therapeutic effects in the future.

## Conclusion

In conclusion, TMB, especially bTMB, is demonstrated to be an independent prognostic factor of OS and PFS in advanced gastric cancer patients receiving first-line chemotherapy. TMB can be considered a representative assessable biomarker to further risk-stratify patients and deserves more attention for the first-line treatment of advanced gastric cancer.

## Data availability statement

The somatic mutation and gene expression RNAseq dataset from TCGA was downloaded from NIH Genomic Data Commons using the GDC downloader tool (https://portal.gdc.cancer.gov/). Any further inquiries can be directed to the corresponding author/s.

## Ethics statement

The studies involving human participants were reviewed and approved by the Institutional Ethics Committee of The Second Affiliated Hospital of Naval Medical University. The patients/participants provided their written informed consent to participate in this study.

## Author contributions

Y-SZ and KL designed the research. X-PD performed the experiments. X-DJ evaluated the clinical data. XH, YL, YW and S-QC collected the data. X-PD wrote the paper. Y-SZ, KL, X-DJ, BL and B-DQ edited the paper. All authors contributed to the article and approved the submitted version.
