# Supplementary material for: Prognostic value of tumor mutation burden in patients with advanced gastric cancer receiving first-line chemotherapy
Source: Front Oncol. 2023 Jan 4;12:1007146. doi: 10.3389/fonc.2022.1007146 (PMC9847361; doi:10.3389/fonc.2022.1007146)
Supplement: Supplementary file 2 [file Table_1.docx]

| **Supplement Table 1** Univariate analyses of clinicopathological factors for OS and PFS in AGC. | | | | | |
| --- | --- | --- | --- | --- | --- |
| Factors | OS | |  | PFS | |
|  | Univariate | |  | Univariate | |
|  | HR (95%CI) | P value |  | HR (95%CI) | P value |
| Gender |  |  |  |  |  |
| Male | 1 |  |  | 1 |  |
| Female | 0.981(0.495-1.942) | 0.955 |  | 0.771(0.4085-1.457) | 0.423 |
| Age |  |  |  |  |  |
| <60 | 1 |  |  | 1 |  |
| ≥60 | 1.216(0.592-2.498) | 0.595 |  | 1.443(0.752-2.769) | 0.27 |
| BMI |  |  |  |  |  |
| 18.5-23.9 | 1 |  |  | 1 |  |
| <18.5 or ≥24 | 1.329(0.675-2.615) | 0.411 |  | 1.312(0.695-2.476) | 0.403 |
| Primary Site |  |  |  |  |  |
| Gastric fundus and body | 1 |  |  | 1 |  |
| Gastric horn and antrum | 0.819(0.453-1.752) | 0.739 |  | 1.045(0.553-1.976) | 0.892 |
| Number of metastatic organs |  |  |  |  |  |
| ＜3 | 1 |  |  | 1 |  |
| ≥3 | 1.898(0.880-4.095) | 0.102 |  | 2.139(1.070-4.277) | 0.032 |
| Previous Surgery |  |  |  |  |  |
| Yes | 1 |  |  |  |  |
| No | 0.976(0.483-1.971) | 0.946 |  | 0.866(0.447-1.677) | 0.669 |
| Local therapy |  |  |  |  |  |
| Yes | 1 |  |  | 1 |  |
| No | 1.537(0.728-3.245) | 0.26 |  | 1.561(0.783-3.099) | 0.204 |
| HER2 immunohistochemistry |  |  |  |  |  |
| Positive | 1 |  |  | 1 |  |
| Negative | 0.903(0.371-2.198) | 0.822 |  | 1.671(0.624-4.476) | 0.307 |
| bTMB |  |  |  |  |  |
| Low | 1 |  |  | 1 |  |
| High | 2.274(1.071-4.827) | 0.032 |  | 2.564(1.184-5.555) | 0.017 |
| tTMB |  |  |  |  |  |
| Low | 1 |  |  | 1 |  |
| High | 3.466(1.360-8.829) | 0.009 |  | 2.908(1.149-7.361) | 0.024 |

Underlined P values are significant

BMI body mass index, HER2 human epidermal growth factor receptor 2, bTMB blood-tested tumor muation burden, tTMB tissue-tesetd tumor mutation burden, OS overall survival, HR hazard ratio, CI confidence interval.
